# Radical Scavenging Activity and Pharmacokinetic Properties of Coumarin–Hydroxybenzohydrazide Hybrids

**DOI:** 10.3390/ijms23010490

**Published:** 2022-01-01

**Authors:** Marko R. Antonijević, Edina H. Avdović, Dušica M. Simijonović, Žiko B. Milanović, Ana D. Amić, Zoran S. Marković

**Affiliations:** 1 Department of Science, Institute for Information Technologies, University of Kragujevac, Jovana Cvijića bb, 34000 Kragujevac, Serbia; mantonijevic@uni.kg.ac.rs (M.R.A.); edina.avdovic@pmf.kg.ac.rs (E.H.A.); dusicachem@kg.ac.rs (D.M.S.); ziko.milanovic@uni.kg.ac.rs (Ž.B.M.); 2 Department of Chemistry, Faculty of Science, University of Kragujevac, Radoja Domanovića 12, 34000 Kragujevac, Serbia; 3 Department of Chemistry, Josip Juraj Strossmayer University of Osijek, Ulica Cara Hadrijana 8A, 31000 Osijek, Croatia; aamic@kemija.unios.hr

**Keywords:** radical scavenging, antioxidants, coumarins, DFT, pharmacokinetics

## Abstract

Free radicals often interact with vital proteins, violating their structure and inhibiting their activity. In previous studies, synthesis, characterisation, and the antioxidative properties of the five different coumarin derivatives have been investigated. In the tests of potential toxicity, all compounds exhibited low toxicity with significant antioxidative potential at the same time. In this paper, the radical scavenging activity of the abovementioned coumarin derivatives towards ten different radical species was investigated. It was found that all investigated compounds show good radical scavenging ability, with results that are in correlation with the results published in the previous study. Three additional mechanisms of radical scavenging activity were investigated. It was found that all three mechanisms are thermodynamically plausible and in competition. Interestingly, it was found that products of the Double Hydrogen Atom Transfer (DHAT) mechanism, a biradical species in triplet spin state, are in some cases more stable than singlet spin state analogues. This unexpected trend can be explained by spin delocalisation over the hydrazide bridge and phenolic part of the molecule with a low probability of spin pairing. Besides radical-scavenging activity, the pharmacokinetic and drug-likeness of the coumarin hybrids were investigated. It was found that they exhibit good membrane and skin permeability and potential interactions with P-450 enzymes. Furthermore, it was found that investigated compounds satisfy all criteria of the drug-likeness tests, suggesting they possess a good preference for being used as potential drugs.

## 1. Introduction

Around 40 years ago, Earl R. Stadtman initiated a research program to investigate the effect of free radicals on enzyme activity and protein turnover and developed methods for monitoring proteins modified by free radical species [1]. His results attracted an increasing number of investigators from various fields. It has been shown that free radicals and reactive oxygen species (ROS) have different roles in living organisms. Due to their nature and diversity, ROS are essential for the regulation of intercellular communication, metabolic processes, immune response to foreign bodies, etc. On the other hand, if they are being produced in excessive amounts, they cause damage to biomolecules in their surroundings. Continuous exposure of the cell to this type of damage causes various disorders that often result in mutations and cell death. Excessive production of ROS is often triggered by external factors such as different types of radiation, the presence of heavy metals, or some other type of changing the normal metabolic pathways in the cell, which causes the state known as oxidative stress (OS). OS is often defined as an imbalance between production and inactivation of ROS by cells antioxidative mechanisms. This imbalance leads to damage of important biomolecules and organs with a potential impact on the whole organism [2]. It is considered that many diseases such as cancer, cardiovascular problems, hypertension, inflammation, as well as neurodegenerative disorders like Alzheimer’s and Parkinson’s disease are a consequence of long-term exposure of the organism to a source of OS [1,2,3,4,5].

In regard to the prevention of ROS induced damage, antioxidants have become an important topic in the last couple of decades [6,7,8]. Antioxidants are compounds that are capable of inactivating free radicals due to their specific molecular structure, making ROS-induced damage light or non-existent. The largest number of these compounds are found in the plant world. There are two main reasons for that. First, due to the reduced ability to change their habitat, plants were forced to find a way (evolve) to fight against the stress induced by external factors in the environment, while animals were able to change the habitat or remove the source of the stress [9,10,11,12].

Coumarin derivates possess numerous therapeutic applications including photochemotherapy, antitumor [13,14,15], and anti-HIV therapy [16,17]. In addition, they are found to be stimulants of the central nervous system [18], antibacterial and anti-inflammatory agents [19], and anti-coagulants [20,21]. Moreover, coumarins are known to be lipid-lowering agents with moderate triglyceride-lowering activity [22]. Hydroxycoumarins show moderate antioxidant activity and have the potential to prevent damage induced by ROS [23]. In our previous study [24], new coumarin–benzohydrazide derivates were synthetized. It was found that they possess significantly better antioxidative potency than their parent molecule, 4-hydroxycoumarin, and excellent antioxidant potency overall. Additionally, newly synthesized hybrids have shown lower potential toxicity in relation to the parent molecule, which makes them good potential antioxidants for use in different branches of industry [24,25]. Due to their potential application in the pharmaceutical or food industry, this paper will discuss their pharmacokinetics and relevant physico-chemical properties.

**Figure 1:**
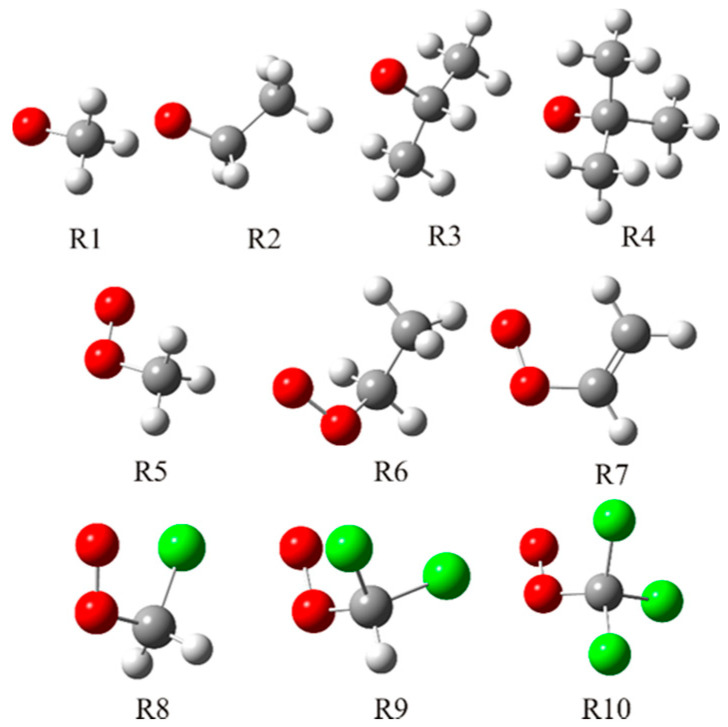
Optimised structures of the investigated free radical species: R1—methoxy radical, R2—ethoxy radical, R3—isopropyloxy radical, R4—*t*–butoxy radical, R5—methyl–peroxy radical, R6—ethyl–peroxy radical, R7—vinyl–peroxy radical, R8—chloromethyl–peroxy radical, R9—dichloromethyl–peroxy radical, and R10—trichloromethyl–peroxy radical.

To examine their antioxidant activity against radicals of different reactivity in detail, a set of three different radical groups has been chosen (Figure 1). The first group contains very reactive alkoxy radicals: methoxy (^•^OCH_3_), ethoxy (^•^OCH_2_CH_3_), isopropyloxy (^•^OCH(CH_3_)_2_), and *t*–butoxy radical (^•^OC(CH_3_)_3_). The second group consists of two alkyl-peroxy (^•^OOCH_3_ and ^•^OOCH_2_CH_3_) and one allyl-peroxy radical (^•^OOCH=CH_2_). The third group consists of chlorinated methyl peroxy radicals. It can be expected, as a consequence of the negative inductive effect of the halogen atom, that these radicals are significantly more reactive than alkyl-peroxy analogues.

### Production of ROS and Metabolism

ROS are often primary and secondary products of the process called autooxidation. In this process, organic molecules are being oxidized by oxygen molecules, not only in living systems but in oils, plastic, rubber, foods, wines, etc. [26]. The process of autooxidation is important because it is often used for the production of certain organic molecules and plays an important role in some enzyme controlled metabolic processes. The presence of the oxygen molecule in biological systems often results in the production of a superoxide radical anion (O_2_^•−^). In general, autooxidation reactions are slow because the O_2_ molecule is not highly reactive, but with the occurrence of superoxide radical anion chain, reaction is started, and the rate of the autooxidation process rapidly increased [26]. These reactions are usually initiated by traces of metal ions, or by some other way of electron excitation from an oxygen molecule. In biological systems, Fe^2+^ initiates superoxide anion production leading to haemoglobin oxidation. However, the haemoglobin complex with Fe(III) produced in process of haemoglobin oxidation, is not able to bind oxygen molecules. It is estimated that around 3% of haemoglobin is being oxidised daily [26].

It is often being said that the most important source of superoxide radical anion is the electron transport chain. However, O_2_^•−^ is necessary for electron transport chain and redox reactions that occur in mitochondria, and in some cases, i.e., in the right concentration, the production of O_2_^•−^ slows down the ageing process of the cell [26]. That being said, inactivation of O_2_^•−^ often leads to the production of ROS, which consequently causes oxidative stress. Biomolecules found in the presence of O_2_^•−^ often produce different alkoxy (RO^•^) and peroxy (ROO^•^) radical species. In addition, carbon-centred radical species (R^•^) (intermediaries in organic reactions, etc.) react with oxygen molecules producing ROO^•^ and subsequently RO^•^, with reaction constant rates higher than 10^9^ M^−1^ s^−1^. Organic peroxides (ROOH), which are usually stable at room temperature, are easily decomposed by heating, UV light exposure, or in the presence of transition metals, producing RO^•^/ROO^•^ [26].

The third group of radicals investigated in this paper is of special significance due to the wide range of toxic effects that these chemical species exhibit when found in the organism. It is well known that carbon tetrachloride (CCl_4_) is toxic to the cells. The main toxic effect of CCl_4_ is manifested in the liver, which causes blockage of the fat extraction causing all sorts of different metabolism failures [27]. When CCl_4_ is found in the presence of the O_2_^•−^, trichloromethyl radical (^•^CCl_3_) is being produced. In an environment with low oxygen concentrations, ^•^CCl_3_ covalently bonds with macromolecules (e.g., cytochrome P–450), which interferes with their biological activities. On the other hand, in oxygen-rich areas, ^•^CCl_3_ easily reacts with the O_2_ molecule, thus forming ^•^OOCCl_3_ that has a significantly wider range of toxic activities [26,27].

Nevertheless, chloroform (CHCl_3_) shows lower toxicity than CCl_4_ [28]. Chloroform is used for various purposes, e.g., as a solvent, as an ingredient in cough medicines, in mouthwashes, etc. [29]. Lower toxicity of chloroform is attributed to a higher energy barrier when producing ^•^CCl_3_. The effect of the number of Cl substituents on the activity of chlorine-substituted methyl-peroxy radicals will be one of the issues addressed in this paper.

## 2. Results and Discussion

**Figure 2:**
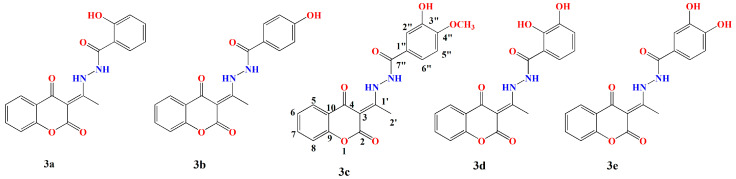
Structures of the investigated compounds (**3a**–**3e**).

Thermodynamic parameters of antioxidative potency, in vitro 2,2-diphenyl-1-picrylhydrazyl radical (DPPH) scavenging activity, and potential toxicity of compounds **3a**–**3e** were investigated in our previous study [24]. It was found that the investigated compounds show moderate to good antioxidative activity. Moreover, in vitro and in silico studies suggested that compounds **3d** and **3e** have exceptionally high antioxidative potency for this class of compounds. Moreover, toxicology prediction showed that investigated compounds often exhibit low toxicity in comparison with the parent molecule, 4–hydroxycoumarin, and some commercially used compounds [24]. Consequently, investigation of their radical scavenging activity is of importance in food and environmental chemistry, especially with radicals containing an organic base, and radicals with halogens as substituents [26,27]. Structures of the investigated compounds are presented in Figure 2.

As mentioned in previous studies [24], a good antioxidative activity of investigated compounds originates from the stability of their radical and anionic species. Namely, the loss of a proton or hydrogen atom from the NH group bonded to C7” enables the formation of a planar chemical species (radical or anion), which allows for better spin and charge delocalisation.

On the other hand, as is well known, the number and position of hydroxyl groups, as well as the methoxy groups on the aromatic ring, play a significant role in stabilizing both the parent molecule and the resulting phenoxy radicals. Therefore, it can be said that these substituents have a great influence on the antioxidant capacity of the investigated compounds [24].

Analysis of the structural parameters of the investigated compounds showed that the presence of an adjacent methoxy group next to the hydroxyl group contributes to better antioxidant properties of the inspected compounds. However, it should be noted that the presence of adjacent hydroxyl groups significantly contributes to better antioxidant properties of these compounds.

The preferred mechanism of radical scavenging activity of investigated compounds with the selected free radicals can be estimated based on thermodynamic parameters of corresponding chemical reactions (9)–(16). The lowest value of a specific thermodynamic parameter defines which mechanism may be dominant. These parameters are presented in Tables S1–S3, for the alkoxy, alkyl-peroxy, and chlorinated methyl-peroxy radicals, respectively, which contains results for the most reactive radical from each group.

It is known that the electronic properties of free radicals have a significant influence on the mechanisms of their action [25]. In order to estimate the influence of both different radicals and their electronic properties, it is necessary to calculate the difference in Gibbs free energy and enthalpy between products and reactants. The obtained values were used as the main criterion for assessing whether the reaction was spontaneous or not. In other words, the thermodynamically favourable reaction pathway is one for which negative values of Gibbs free energies are obtained.

The calculated values for the Gibbs free energy change for all investigated antioxidant mechanisms in polar and nonpolar solvents are shown in Tables S1–S3. It should be emphasized that the obtained results, using Equations (9)–(16a,b), are mutually consistent.

The obtained results indicate that SET–PT mechanism is not thermodynamically possible due to the fact that Δ*_r_G*_SET_ values, which describe the first step of this mechanism are positive, which indicates endergonic reaction. A similar picture was obtained for the reactions that follow the RAF mechanical path. Despite the fact that positive values for Δ*_r_G*_RAF_ are often obtained, this mechanistic path must not be neglected. Especially if it is known that small positive values (<40 kJ/mol) mean that the appropriate reaction path should be taken into account. For all positions that the Fukui index predicts to be reactive, the values of thermodynamic parameters were calculated and given in the Supplementary Material (Tables S4–S13).

Analysis of the values given in Tables S1–S3 shows that the thermodynamic parameters describing HAT as well as the first step of the SPLET mechanism, Δ*_r_G*_HAT_ and Δ*_r_G*_SPL_, are negative, indicating that both mechanisms are thermodynamically possible. However, the reaction of hydrogen atom transfer is less exergonic than proton abstraction, which makes SPLET the predominant mechanistic pathway.

### 2.1. SPLET and HAT Mechanisms

As mentioned in the previous section, the most probable radical-scavenging reaction pathway is SPLET. Nevertheless, careful examination of the results from Table S2 reveals an interesting trend concerning the Δ*_r_G*_SPL_ values. As expected from the structures and previous studies, the highest radical scavenging activity is expressed by compounds **3d** and **3e** [24]. However, compound **3a** shows unexpectedly low Δ*_r_G*_SPL_ values in the C7”–NH position, which indicates better radical scavenging activity of this compound compared even with the **3e**, which has one OH group more. This is a consequence of the additional stabilisation of the anion by the intramolecular hydrogen bond forming between the carbonyl oxygen in the C7” position and the OH group in the position C2”. This allows a better delocalisation of the negative charge from the nitrogen throughout pseudo-ring formed by an intermolecular hydrogen bond. This type of intramolecular hydrogen bond also exists in the case of the anion formed in the C7”–NH position of **3d**. Compounds **3b**, **3c**, and **3e** show slightly lower radical scavenging activity due to the fact they lack this type of stabilisation. A more detailed explanation of this phenomenon is given in our previous research paper [24].

According to the obtained results, radical scavenging activity of the investigated compounds descends in the following order: **3d**, **3a**, **3e**, **3b**, and **3c**. If the abstraction of protons from the C7”–NH position is prevented due to steric hindrance, then it can take place via OH groups on the aromatic ring. In that scenario, radical scavenging potential descends in the following order: **3e**, **3b**, **3d**, **3c**, and **3a**.

Although SPLET is the dominant mechanistic pathway and Δ*_r_G*_SPL_ values are lower in benzene than in water, in an acidic medium or aprotic, a nonpolar solvent in which anion formation is not possible, the HAT mechanism should be considered. By careful examination of the obtained results, it is possible to see that Δ*_r_G*_HAT_ values for the position C7”–NH are quite similar, regardless of the solvent or number and the position of OH groups of the phenyl/catechol part of the molecule. Generally speaking, the C7”–NH position is the most preferred one for all investigated compounds, except for **3e**. The formation of stable nitrogen radicals at nitrogen bound to C7” in all compounds is a consequence of the planarity of the obtained radicals which allows good spin delocalization via the coumarin base and the phenolic/catechol part of the test compound. The only compound **3e** has two adjacent OH groups in the para and meta positions. This allows good spin delocalization when a hydrogen atom is abstracted from one of its. This is due to the presence of a catechol structure on the aromatic ring, which is known to have a significant effect in improving radical scavenging activity [30].

This fact makes these positions thermodynamically equal, and sometimes more desirable in relation to the position C7”–NH. Especially if the radicals with which investigated compound reacts are more voluminous (Table 1) because then steric hindrance comes to the fore which reduce the possibility of abstracting hydrogen atoms from C7”–NH position. The descending order of reactivity following the HAT mechanism will be **3e**, **3d**, **3c**, **3b**, and **3a**.

### 2.2. Radical Scavenging Activity in Regard to the Radical’s Structure

#### 2.2.1. Alkoxy and Peroxy Radicals

According to the results from Table S1, contrary to expectations, the number of methyl groups does not have a significant effect on the radical scavenging activity of the investigated compounds against alkoxy radicals. Differences in Δ*_r_G_SPL_* between methoxy, ethoxy, and isopropyloxy radicals in water are almost non-existent. The *t*-butoxy radical shows values that are around 4 kJ/mol lower than for the other three radicals, consistently, in all positions. In benzene, because there is no anion stabilisation by the solvent, differences in Δ*_r_G_SPL_* values are more pronounced. The presence of an additional –CH_3_ group leads to the increase in Δ*_r_G_SPL_* values, as a consequence of the positive inductive effect of methyl groups. Consequently, Δ*_r_G_HAT_* values in both solvents decrease in the following order: ^•^OCH_2_CH_3_ < ^•^OCH_3_ < ^•^OCH(CH_3_)_2_ < ^•^OC(CH_3_)_3_.

When it comes to peroxyl radicals, in benzene there is no significant difference between methyl-peroxy and ethyl-peroxy radicals in neither Δ*_r_G_HAT_* nor Δ*_r_G_SPL_* values. However, in water anions are stabilised by solvent molecules, Δ*_r_G_SPL_* values are lower for approximately 5 kJ/mol in the presence of the additional methyl group. Moreover, there is a difference in Δ*_r_G_HAT_* values in the water around 1 kJ/mol in favour of ethyl-peroxy radical. On the other hand, according to the results from Table S2, the presence of a double bond lowers the Δ*_r_G_HAT_* values simultaneously increasing the values of Δ*_r_G_SPL_*. The reason is that in vinyl-peroxy radical unpaired electrons can be delocalised over the double bond. Thanks to the inductive and resonance effect the charge on the peroxy-vinyl anion is better delocalised (Figure S1), which lowers the ability of the proton abstraction from the antioxidant molecule. Expectedly, these differences are more pronounced in the non-polar medium. Because of the different effects of substituents on radical and anion stability, there is a significant difference in the order of the radical scavenging activity regarding the mechanistic pathway. Order of reactivity in radical-scavenging reactions that follow SPLET mechanistic pathway decreases as follows: ^•^OOCH_3_CH_2_ > ^•^OOCH_3_ > ^•^OOCH=CH_2_. Order of reactivity for the HAT mechanism would be ^•^OOCH=CH_2_ > ^•^OOCH_3_CH_2_ ≥ ^•^OOCH_3_.

#### 2.2.2. Chlorinated Methyl-Peroxy Radicals

It was concluded, in the last subsection, that introducing an electron-rich substituent with the negative inductive and positive resonance effect leads to the decrease in Δ*_r_G*_HAT_ and increase in Δ*_r_G*_SPL_ values. A similar conclusion can emerge from the results presented in Table S3. In comparison to the results obtained for methyl-peroxy radical (Table S2), the substitution of one of the hydrogen atoms in methyl group with chlorine atom will lower the Δ*_r_G*_HAT_, simultaneously increasing Δ*_r_G*_SPL_ values, as it was the case for the double bond in vinyl-peroxy radical. However, the influence of substituents gradually decreases with the number of already substituted hydrogen atoms. It means that the difference in the Δ*_r_G*_HAT_ values between ^•^OOCH_3_ and ^•^OOCH_2_Cl is around 25 kJ/mol, between ^•^OOCH_2_Cl and ^•^OOCHCl_2_ is a little over 10 kJ/mol, and between ^•^OOCHCl_2_ and ^•^OOCCl_3_ is less than 3 kJ/mol.

The difference in Δ*_r_G*_SPL_ values is even more obvious and it depends on the solvent. In water, between ^•^OOCH_3_ and ^•^OOCH_2_Cl difference is around 40 kJ/mol, while the difference between ^•^OOCH_2_Cl and ^•^OOCHCl_2_, as well as between ^•^OOCHCl_2_ and ^•^OOCCl_3_, is about 20 kJ/mol. Meanwhile, in benzene, the difference between ^•^OOCH_3_ and ^•^OOCH_2_Cl is around 55 kJ/mol, between ^•^OOCH_2_Cl and ^•^OOCHCl_2_, as well as ^•^OOCHCl_2_ and ^•^OOCCl_3_, is about 25 kJ/mol.

##### SPLET and HAT as Competitive Mechanisms in Trichloromethyl-Peroxy Radicals Inactivation

The fact that Δ*_r_G*_HAT_ values are decreasing and Δ*_r_G*_SPL_ values are being increased, raises a question about the dominant mechanism in the case of ^•^OOCCl_3_, where these parameters overlap. This means that SPLET and HAT mechanisms in the case of trichloromethyl-peroxy radical are in competition, and the dominant mechanistic pathway will depend on the even slightest changes in the environment or structure of the antioxidative molecule. In water, for compounds **3a** and **3d**, the preferable mechanism is SPLET but only when C7”–NH position is considered. Because ^•^OOCCl_3_ is a voluminous radical it could be expected that this position will not be easily accessible, and a large percentage of reactions would follow the HAT mechanism. According to the thermodynamic parameters for compounds **3b**, **3c**, and **3e**, HAT will be the preferred mechanism in all positions. In benzene, according to the thermodynamic parameters, SPLET is the dominant mechanism in most cases.

It is important to emphasize that alkoxy radicals are the most reactive out of the three investigated radical groups. That being said, the activity of the peroxy radicals, in regard to chlorinated methyl-peroxy radicals, depends on the conditions that determine the reaction pathway. If reaction follows the HAT mechanistic pathway, chlorinated methyl-peroxy radicals will be more reactive towards investigated compounds because of the better spin delocalization. On the other hand, if the reaction follows SPLET mechanism peroxy radicals will react more rapidly.

### 2.3. RAF Mechanism

It was mentioned earlier that in the case of the **3a** and **3d**, the increased spin and charge delocalisation provided by pseudo-ring formation due to the hydrogen bond between carbonyl group in position C7”, and hydroxy group in position C2”, indicates higher radical scavenging potency. This has one more interesting trend for a consequence. Namely, results obtained by the Fukui index, for all investigated compounds indicated that the favoured position was C7 of a coumarin moiety. However, in the case of **3a** and **3d**, potentially good reactivity towards free radical species was predicted for additional two positions, C6” and C5”. Moreover, the reactivity of the C7 position was increased in comparison to the other three investigated compounds. Values of the Fukui indexes in various positions are presented in the Supplementary material (Figure S2).

For many molecules of organic antioxidants, the RAF is one of the most probable mechanistic pathways for removing free radicals, especially those with π-conjunction systems, as is the case with the researched compounds. Unfortunately, in the case of the investigated compounds, the values of Δ*_r_G*_RAF_ indicate that the reactions that follow this mechanism are in most cases endergonic.

Analysis of free energy values for reactions of alkoxy radicals with the investigated compounds reveals that all reactions are slightly exergonic. By comparing these values with the corresponding ones for HAT and SPL, it is clear that they are dominant, compared to RAF values. That being said, the reactivity decreases with the increase in the number of methyl substituents on radical species and increases with the number of electronegative substituents. This can be explained by the examination of spin delocalisation on radical species presented in Figure S4. The highest spin density is presented on the oxygen of trichloromethyl radical followed by di- and chloromethyl radicals, which indicates that the order of reactivity, starting with trichloromethyl radical, as most reactive. Moreover, when the reaction proceeds by the RAF mechanism, sp^3^ hybridized carbon atom is formed. Its formation leads to the interruption of the delocalization of the π-electron in the aromatic ring, and thus to the disruption of planarity. The adduct thus obtained is less stable, which is reflected in less exergonic reaction, and the RAF mechanism itself is a less favourable mechanical pathway.

### 2.4. Two-Step Mechanisms of Radical Scavenging Activity

The products obtained after the radical scavenging process by either HAT, SET-PT, or RAF mechanism are less reactive free radicals than the starting ones. Although the resulting free radicals are less reactive, they are still very reactive species. Therefore, their reactions with another free radical species were examined below. For this purpose, the DHAT, RAF-HAT, HAT-RAF, and HAT-RC mechanisms were tested. Only the most reactive radical species from the three radical groups were considered. In addition, based on thermodynamic parameters, the RAF mechanism is a much less likely reaction pathway in comparison to HAT. For these reasons, the RAF-HAT mechanism is excluded from further analysis in this paper.

#### 2.4.1. Double HAT Mechanism

By following the DHAT mechanistic pathway, radical species derived from the antioxidant molecule by HAT or even SPLET mechanism can react with another of the investigated free radicals via the HAT mechanism. This leads to the formation of biradical species that can be stabilized by spin pairing and forming neutral chemical species. It is well known that most compounds are usually more stable in the singlet state. However, some compounds, and/or their respective chemical species are more stable in triplet than singlet spin state. For these reasons, it is necessary to consider both possibilities in the further analysis of the obtained results [31,32]. The obtained thermodynamic parameters are presented in Table 1.

**Figure 3:**
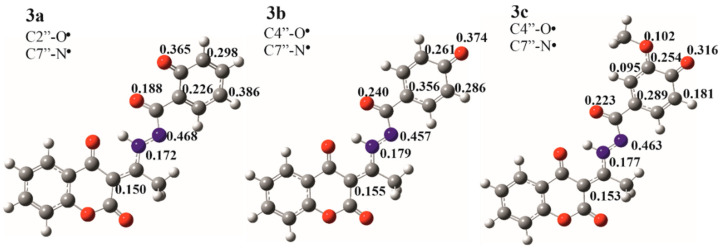
NBO spin delocalisation of **3a**, **3b**, and **3c** biradicals obtained through the DHAT mechanism.

According to the results presented in Table 1, reactions following DHAT are often exergonic which makes it a thermodynamically plausible, and in some cases favourable mechanistic pathway. Compounds **3a**, **3b**, and **3c** show lower Δ*_r_G_DHAT_* when the reaction products are in the triplet than in the singlet spin state, which is contrary to expectations because this phenomenon is usually associated with large aromatic molecules [31,32]. This can be explained by spin delocalisation. As can be seen from Figure 3, spins are usually delocalised over two areas of the molecule. First unpaired electron obtained by radical forming in position C7”–N^•^ is delocalised over the hydrazide bridge of the investigated molecule, while unpaired electron from –OH group is often delocalised on phenol/catechol part of the molecule.

This kind of spin delocalization indicates the high stability of these biradicals in the triplet spin state. The high stability of the biradicals in triplet over singlet spin state is also confirmed by the differences in energies of HOMOs presented in Table S14. Generally, organic conjugated molecules with strong electron donor–acceptor coplanar skeleton have a good electron delocalization [32].

**Figure 4:**
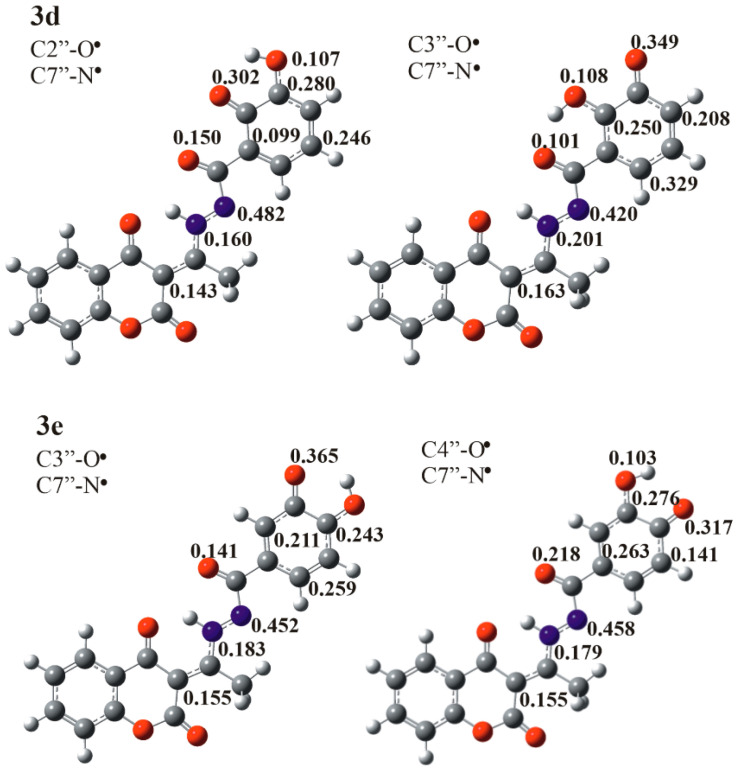
NBO spin delocalisation of **3d** and **3e** biradicals obtained through the DHAT mechanism.

A similar situation is observed in the case of compound **3d** when radicals are formed in positions C7”–NH and C2”–OH, and in **3e** with radicals are formed in C7”–NH and either OH group (Figure 4). The expected conclusion would be that position C7”–NH and C3”–OH in **3d** compound would follow this trend. However, in a singlet spin state, hydrogen atom transfer from hydroxy to the carbonyl group additionally stabilizes the molecule.

The lower stability in triplet spin states can be explained by spin delocalization. The spin density decreases on the hydrazide bridge and the carbonyl oxygen and moves towards the catechol ring, which leads to the pairing of electrons, that is, the singlet state.

Molecules are generally more stable and less reactive in a singlet, which means that **3d** and **3e** are better free radical scavengers. This hypothesis is confirmed by significantly lower values of Δ*_r_G*_DHAT_ (Table 1), as well as the values of Gibbs free energies, which are given in Table S16. As expected, reactions from the second step of the DHAT mechanism are solvent depended and favour the polar environment, especially in the singlet spin state. Moreover, it is important to notice that the most reactive radical species, regarding a DHAT mechanistic pathway, are alkoxy followed by chlorinated methyl peroxy and vinyl peroxy radicals, which follows a previously established trend.

#### 2.4.2. HAT–RAF Mechanism

Both HAT and SPLET result in the production of the radical species derived from the antioxidant molecule. RAF is another potential mechanistic pathway for further removal of free radicals by means of radical of investigated antioxidant. In these reactions, the carbon atom to which the free radical attaches is rehybridized from sp^2^ to sp^3^. The intermediates obtained in this way has an interrupted planar structure, especially with voluminous radicals. This results in a reduced possibility of delocalization of the unpaired electron, and thus a more unstable intermediate is obtained.

The resulting adducts can be in the triplet (biradical) or singlet state. In the first case, all reactions are endergonic (Table S14), while in the second one, they are often exergonic. Based on this, we can conclude that the HAT-RAF mechanism should be taken into account in free radical scavenging reactions.

**Scheme 1:**
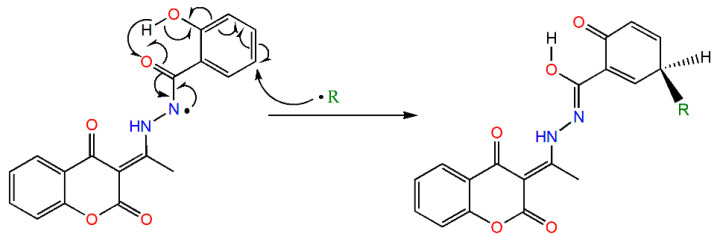
Intramolecular hydrogen transfer as a spin stabilisation mechanism.

Interesting results were obtained for compounds **3d** and **3a.** According to the values in Tables S14 and S15, the HAT-RAF mechanism can be considered the dominant mechanism in positions C5” for both compounds. This is due to the hydrogen rearrangement from the C2”–OH group to carbonyl oxygen, which allows a stable molecule to be obtained. This process is presented in the following scheme (Scheme 1):

Optimised structures of the compounds stabilised by the hydrogen atom rearrangement from C2”–OH to C7”=O are presented in Figure S3. This allows unpaired electrons to be found on the phenolic/catechol part of the molecule, and for the electrons to pair and form a stable molecule. It should be noted that based on thermodynamic parameters, DHAT is a more likely mechanism in all other positions, except for the HAT-RAF in position C5”.

It should be noted that in the case of adduct formation on the coumarin part of the molecule in position C7, a different distribution of spin density is observed (Figure S5). It can be easily seen that most of the spin density is delocalized via the coumarin part of the molecule, while the rest is delocalized via the hydrazide bridge.

It is important to emphasize that spin delocalisation depends on the radical specie as well, and some exceptions are to be expected, especially for radicals with a highly positive inductive effect like *t*-butoxy radical, whereas radicals like trichloromethyl peroxy radicals are less prone to spin pairing effect due to highly negative inductive effect.

#### 2.4.3. Radical Coupling Mechanism

**Scheme 2:**
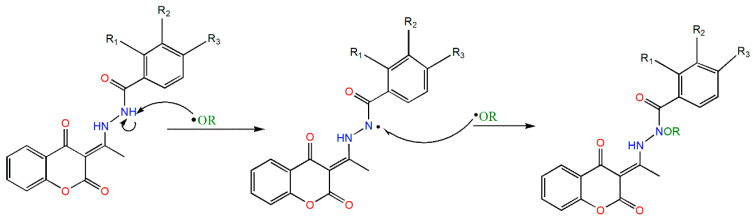
HAT-RC mechanistic pathway for compounds **3a**–**3e**.

A special case of the HAT-RAF mechanism is when a free radical forms an adduct on the atom with the highest spin density. It is usually an atom on which a radical is formed either by the HAT or SPLET mechanism. The general scheme of the HAT-RC mechanism is presented in the following scheme (Scheme 2):

As expected, because of the same position of this mechanism happening and similarity in the structures of investigated compounds, values for ∆*_r_G*_HAT-RC_ values are very similar for all investigated compounds. Results presented in Table S20 indicate that HAT-RC is more favourable than the HAT-RAF mechanism in all cases except for when adduct is formed in position C5” of compounds **3a** and **3d**. Moreover, HAT-RC is in competition with DHAT mechanistic pathway and is even more favourable in the case of **3a**, **3b**, and **3c** for all investigated radicals. In the case of **3d** and **3e**, because of the spin stabilisation, DHAT is a preferred mechanistic pathway.

### 2.5. In Silico Evaluation of Pharmacokinetics and Drug-Likeness

#### 2.5.1. Pharmacokinetics

In order to predict the chemical behaviour of the investigated compounds, besides the potential toxicology obtained in the earlier studies [24], in silico pharmacokinetics and drug-likeness were evaluated. In order to obtain parameters that describe pharmacokinetics and drug-likeness, investigated compounds were subjected to the analysis run by the SwissADME web server, and the obtained results were presented in Table 2. For confirmation of the results obtained by implementation of SwissADME server, ADMETlab 2.0 server was used, and the results are presented in Tables S18 and S19. It was found that results for SwissADME and ADMETlab 2.0 server are in direct correlation.

As can be seen from the obtained results presented in Table 2, all tested compounds show potentially high gastrointestinal absorption (GI) and low blood–brain barrier permeability (BBB). At the same time, it was found that compound **3a** has the lowest while the **3e** has the highest predicted skin permeability according to the value of skin permeation coefficient *Kp*. Additionally, it is well known that coumarin molecules are easily transported by albumin molecules to the cell and potentially easily distributed throughout the organism, without crossing the brain barrier.

Coumarin derivates interact with active sites of different proteins and enzymes. Cytochrome P450 enzymes are essential for the metabolism of many medications. As it was mentioned in the introduction part of this paper, these enzymes often are a target of some chlorinated-methylperoxy radicals. Although this class consists of a large number of different isoforms of P450 enzymes, six of them metabolize 90% of known drugs. As results presented in Table 2 show, all investigated compounds are potential inhibitors of CYP1A2, and almost all of them (except **3e**) are the inhibitors of the CYP2C9. Even though investigated compounds do not show inhibitory activity towards CYP2C19 and CYP2D6, it is important to notice that compounds **3c** and **3d** have the potential to inhibit CYP3A4, which is one of the most important P450 enzymes. These results indicate that if these compounds are used as potential drugs, their activity will be more pronounced in patients without CYP1A2, CYP2C9, and CYP3A4 in their genotype. However, compounds **3a**–**3e** can be used as inhibitors of these P450 enzymes in order to enhance the effect of some other drug affected by the enzyme’s function. For example, warfarin is inactivated by the CYP2C9, and the presence of the investigated compounds **3a**–**3d** can potentially increase the concentration of warfarin in the blood, which can lead to the increased bleeding of the patient. At the same time, if a patient needs a higher concentration of warfarin in blood, for prevention of the heart attack, for example, the same compound can show a positive effect by binding to CYP2C9 and allowing for elevation of warfarin concentrations. These effects are dependent on the difference in binding energies between the enzyme, investigated compound, and a specific drug molecule, which can be further evaluated by molecular docking and molecular dynamic simulations for every drug specifically.

#### 2.5.2. Drug-Likeness Prediction

By examination of the great number of parameters, the SwissADME server can determine the drug-likeness with the five different approaches following Lipinski’s, Ghose’s, Egan’s, Veber’s, and Muegge’s rules. Additionally, ADME analysis performed by implementation of ADMETlab 2.0 web service was used as confirmation of the results obtained by the SwissADME server. All investigated compounds were subjected to this analysis and all five compounds have met all drug-likeness criteria; therefore, they can be considered druglike structures. Results of this analysis, as well as the bioavailability of investigated compounds, can be found in Table S17.

## 3. Materials and Methods

All calculations were carried out by the Gaussian09 software package [33]. For optimization of neutral molecules, the corresponding radicals, anions, and radical cations density functional theory (DFT) method M06–2X with 6–311++G(d,p) was used. This theoretical model is suitable for thermodynamic and kinetic investigation of reaction mechanisms of examined compounds with ROS [34,35]. All investigated structures were optimized in polar and nonpolar media without any geometrical constraints. The solvent effect was included by the Conductor-like Polarizable Continuum Model (CPCM) [36].

Four antioxidant mechanisms were selected for the evaluation of the radical scavenging activity: Hydrogen Atom Transfer (HAT), Single-Electron Transfer followed by Proton Transfer (SET–PT), Sequential Proton Loss Electron Transfer (SPLET), and Radical Adduct Formation (RAF) [37,38,39,40]. The HAT mechanism is characterised by a rapid, one-step abstraction of a hydrogen atom from the antioxidant molecule by the free radical species (Equation (1)). SET–PT mechanism consists of two consequential steps. In the first step, the electron is transferred to the free radical from the antioxidant molecule and the radical cation is formed (Equation (2)), while the second step represents the proton transfer from the radical cation to radical (Equation (3)). SPLET mechanism is also a two-step process where the order of the particles exchange is reversed in regard to SET–PT. In the first step, an anion is being formed from the antioxidant molecule, and by heterolytic dissociation, electron transfer is performed in the second step (Equations (4) and (5)). Lastly, described by Equation (6) is the RAF mechanism, in which an adduct is formed between the radical and the antiradical species.

Ar–OH + RO^•^ → Ar–O^•^ + ROH (1)

Ar–OH + RO^•^ → Ar–OH^•+^ + RO^−^ (2)

Ar–OH^•+^ + RO^−^ → Ar–O^•^ + ROH (3)

Ar–OH + RO^−^ → Ar–O^−^ + ROH (4)

Ar–O^−^ + RO^•^ → Ar–O^•^ + RO^−^ (5)

Ar–OH + RO^•^ → [RO–Ar–OH]^•^ (6)

The notations Ar–OH, Ar–OH^•+^, Ar–OH^•^, and Ar–O^−^, represent neutral specie, radical cation, radical, and anion of the radical scavenging agent, respectively.

Additional mechanisms of the radical-scavenging activity in which radical or adduct obtained, namely, Double Hydrogen Atom Transfer (DHAT), HAT-RAF, and HAT followed by radical–radical coupling (HAT-RC) by the abovementioned mechanisms are presented by the following equations (Equations (7a), (7b), (8a), (8b) and (9)):

Ar–O^•^ + RO^•^ → Ar=O + ROH (7a)

Ar–O^•^ + RO^•^ → ^•^Ar–O^•^ + ROH (7b)

Ar–O^•^ + RO^•^ → [RO–Ar–OH] (8a)

Ar–O^•^ + RO^•^ → ^•^[RO–Ar–OH]^•^ (8b)

Ar–O^•^ + RO^•^ → [RO–Ar–OH] (9)

Equations (7a), (7b) and (8a), (8b) representing the DHAT and HAT-RAF mechanisms in singlet/triplet, respectively, and Equation (9) representing HAT-RC mechanism.

Thermodynamic parameters that describe radical scavenging activity of investigated compounds are calculated according to the following equations (Equations (10)–(15)):

Δ*_r_G_HAT_* = *G*(Ar–O^•^) + *G*(ROH) − *G*(Ar–OH) − *G*(RO^•^) (10)

Δ*_r_G_SET_* = *G*(Ar–OH^•+^) + *G*(RO^−^) − *G*(Ar–OH) − *G*(RO^•^) (11)

Δ*_r_G_PT_* = *G*(Ar–O^•^) + *G*(ROH) − *G*(Ar–OH^•+^) − *G*(RO^−^) (12)

Δ*_r_G_SPL_* = *G*(Ar–O^−^) + *G*(ROH) − *G*(Ar–OH) − *G*(RO^−^) (13)

Δ*_r_G_ET_* = *G*(Ar–O^•^) + *G*(RO^−^) − *G*(Ar–O^−^) − *G*(RO^•^) (14)

Δ*_r_G_RAF_* = *G*([RO–Ar–OH]^•^) − *G*(Ar–OH) − *G*(RO^•^) (15)

Thermodynamic parameters describing the DHAT and HAT-RAF mechanisms are presented in Equations (16a)–(17b) in singlet and triplet spin state, as well as HAT-RC presented in Equation (18).

Δ*_r_G_DHAT_* = *G*(Ar=O) + *G*(ROH) − *G*(Ar–O^•^) − *G*(RO^•^) (16a)

Δ*_r_G_DHAT_* = *G*(^•^Ar–O^•^) + *G*(ROH) − *G*(Ar–O^•^) − *G*(RO^•^) (16b)

Δ*_r_G_HRAF_* = *G*([RO–Ar–OH]) − *G*(Ar–O^•^) − *G*(RO^•^) (17a)

Δ*_r_G_HRAF_* = *G*(^•^[RO–Ar–OH]^•^) − *G*(Ar–O^•^) − *G*(RO^•^) (17b)

Δ*_r_G_HAR-RC_* = *G*(RO–Ar–OH) − *G*(Ar–O^•^) − *G*(RO^•^) (18)

The obtained results will be entered into the database in order to form a tool that will be able to select which molecular structures are prone to be antioxidants based on the obtained thermodynamic parameters, using artificial intelligence [41].

### 3.1. Fukui Functions

As proposed by Yang and Parr, the Fukui functions represent a differential change in electron density when the number of electrons in the molecule has been changed [42]. These functions can be used for the prediction of the most probable reaction site for the radical attack and are calculated by the following equation:

(19) $$f^{0}=\frac{\rho_{N+1\;}+\rho_{N-1}}{2}$$

where $\rho_{N+1\;}$ represents the radical anion $\rho_{N-1\;}$ represents the radical cation of the neutral molecule $\rho_{N\;}$. When Equation (19) is integrated for the individual atoms, Fukui functions for individual atoms are obtained and an active site is calculated as the difference between the charge on atom A of cationic $\left(q_{N-1}^{A}\right)$ and anionic $\left(q_{N+1}^{A}\right)$ species (Equation (20)):

(20) $$f_{A}^{0}=\frac{q_{N-1}^{A}-q_{N+1}^{A}}{2}$$

### 3.2. In Silico Analysis of Pharmacokinetic Parameters and Drug-Likeness

In order to evaluate absorption, distribution, metabolism, and excretion (ADME) of the investigated compounds, as well as other pharmacokinetic and physicochemical properties, SwissADME server, developed by the Swiss Institute of Bioinformatics, as well as ADMETlab 2.0 server, was used [43,44].

## 4. Conclusions

In our previous study, coumarin–benzohydrazide hybrids **3a**–**3e** showed moderate to excellent antioxidative activity and low potential toxicity. In this paper, compounds **3a**–**3e** were subjected to investigation of the mechanisms of their radical scavenging activity, as well as their pharmacokinetic properties and drug-likeness. It was found that all investigated compounds exhibit good radical scavenging ability, especially in the case of alkoxy radicals. SPLET and HAT were found to be the operative mechanisms of radical scavenging. It was found that both mechanisms are mutually competitive and dependent on the solvent and the radical species being inactivated. On the other hand, based on thermodynamic parameters, the SET-PT and RAF mechanisms were proved to be non-operational. Besides the usual mechanisms of radical scavenging activity, two additional mechanisms were investigated. In reactions following the DHAT mechanistic pathway, it was interesting to find that in some cases products in triplet spin state are slightly more stable than in singlet spin state. However, compounds **3d** and **3e** were found to have more stable products in singlet spin state, which indicates the higher stability of obtained products and better antioxidative activity of those two compounds in regard to **3a**–**3c**, which is in correlation to the results obtained in our previous study. At the same time, in reactions following the HAT–RAF mechanism, interesting stabilization of the obtained adduct was found when a reaction happens in position C5”. Namely, in order to stabilize spin, a hydrogen atom from the OH group in position C2” is transferred to the carbonyl group in position C7”, making the adduct stable and HAT-RAF more competitive compared to DHAT.

The pharmacokinetics and drug-likeness study was performed by SwissADME and ADMETlab 2.0 servers. The obtained results were in correlation and indicate the good overall potential of investigated compounds to be used as potential drugs.

## Figures and Tables

**Table 1:** Thermodynamic parameters describing the second step of the DHAT mechanism with products in singlet and triplet spin states.

Compound		Δ*_r_G_DBDE_*					
		(CH_3_)_3_CO^•^		CH_2_=CHOO^•^		Cl_3_COO^•^	
Singlet State		Water	Benzene	Water	Benzene	Water	Benzene
**3a**	C2”–O^•^ and C7”–N^•^	4	43	77	118	56	97
**3b**	C4”–O^•^ and C7”–N^•^	−28	0	45	75	24	54
**3c**	C4”–O^•^ and C7”–N^•^	−71	−19	2	55	−19	35
**3d**	C2”–O^•^ and C7”–N^•^	−12	27	61	102	40	81
	C3”–O^•^ and C7”–N^•^	−132	−102	−59	−27	−80	−47
	C2”–O^•^ and C3”–O^•^	−139	−96	−66	−21	−87	−42
**3e**	C3”–O^•^ and C7”–N^•^	−36	2	38	77	16	56
	C4”–O^•^ and C7”–N^•^	−63	−33	11	41	−11	21
	C3”–O^•^ and C4”–O^•^	−144	−107	−71	−32	−92	−52
**Compound**		**Δ*_r_G_DBDE_***					
		**(CH_3_)_3_CO^•^**		**CH_2_=CHOO^•^**		**Cl_3_COO^•^**	
**Triplet State**		**Water**	**Benzene**	**Water**	**Benzene**	**Water**	**Benzene**
**3a**	C2”–O^•^ and C7”–N^•^	−39	−9	34	65	13	45
**3b**	C4”–O^•^ and C7”–N^•^	−58	−35	15	40	−7	19
**3c**	C4”–O^•^ and C7”–N^•^	−94	−46	−21	29	−42	8
**3d**	C2”–O^•^ and C7”–N^•^	−74	−44	-1	30	−22	10
	C3”–O^•^ and C7”–N^•^	−83	−55	−10	20	−32	−1
	C2”–O^•^ and C3”–O^•^	20	23	94	98	72	77
**3e**	C3”–O^•^ and C7”–N^•^	−91	−67	−18	7	−39	−13
	C4”–O^•^ and C7”–N^•^	−89	−66	−16	9	−38	−12
	C3”–O^•^ and C4”–O^•^	17	31	90	105	68	85

**Table 2:** Predicted pharmacokinetic parameters of investigated compounds by SwissADME server.

Compound	GI	BBB	Log*Kp* (cm/s)	P–gp Substrate	CYP1A2 Inhibitor	CYP2C19 Inhibitor	CYP2C9 Inhibitor	CYP2D6 Inhibitor	CYP3A4 Inhibitor
**3a**	High	No	−5.59	No	Yes	No	Yes	No	No
**3b**	High	No	−5.98	No	Yes	No	Yes	No	No
**3c**	High	No	−6.18	No	Yes	No	Yes	No	Yes
**3d**	High	No	−5.94	No	Yes	No	Yes	No	Yes
**3e**	High	No	−6.33	No	Yes	No	No	No	No

## Data Availability

Data is contained within the article and Supplementary Material.

## Supplementary Materials

The following supporting information can be downloaded at: https://www.mdpi.com/article/10.3390/ijms23010490/s1.
