# Supplementary material for: Radical Scavenging Activity and Pharmacokinetic Properties of Coumarin–Hydroxybenzohydrazide Hybrids
Source: Int J Mol Sci. 2022 Jan 1;23(1):490. doi: 10.3390/ijms23010490 (PMC8745304; doi:10.3390/ijms23010490)
Supplement: Supplementary file 1 [file ijms-23-00490-s001.zip › ijms-1517320-supplementary.pdf]

Supplementary Material

Radical scavenging activity and pharmacokinetic properties of coumarin-hydroxybenzohydrazide hybrids

Marko R. Antonijević[1,2] ,Edina H. Avdović[1], Dušica M. Simijonović[1], Žiko Milanović[1], Ana Amić[3], Zoran S. Marković[1*]

[1]University of Kragujevac, Institute for Information Technologies, Department of Science, Jovana Cvijića bb, 34000 Kragujevac, Serbia
[2]University of Kragujevac, Faculty of Science, Department of Chemistry, Radoja Domanovića 12, 34000 Kragujevac, Serbia
[3]Department of Chemistry, Josip Juraj Strossmayer University of Osijek, Ulica cara Hadrijana 8A, 31000 Osijek, Croatia; aamic@kemija.unios.hr
**Correspondence: e-mail address: zmarkovic@uni.kg.ac.rs ; phone number: +38134/610-01-95*

Table S1: Thermodynamic parameters for radical scavenging activity of investigated compounds with alkoxy radicals (kJ mol^{-1}).

With **alkoxy** radicals		HAT	SET-PT		SPLET		HAT	SET-PT		SPLET	
		$\Delta_r G_{HAT}$	$\Delta_r G_{SET}$	$\Delta_r G_{PT}$	$\Delta_r G_{SPL}$	$\Delta_r G_{ET}$	$\Delta_r G_{HAT}$	$\Delta_r G_{SET}$	$\Delta_r G_{PT}$	$\Delta_r G_{SPL}$	$\Delta_r G_{ET}$
		**Water**					**Benzene**				
**•OCH$_3$**											
**3a**	C7"-NH	**-81**	225	-307	**-166**	85	**-86**	417	-504	**-229**	143
	C2"-OH	**-44**		-270	**-92**	48	**-32**		-450	**-133**	101
**3b**	C7"-NH	**-80**	214	-294	**-146**	66	**-86**	403	-489	**-204**	118
	C4"-OH	**-53**		-267	**-118**	64	**-51**		-454	**-171**	120
**3c**	C7"-NH	**-81**	219	-278	**-143**	64	**-86**	402	-488	**-203**	117
	C4"-OH	**-61**		-280	**-101**	39	**-57**		-459	**-157**	100
**3d**	C7"-NH	**-79**	180	-259	**-168**	89	**-83**	424	-506	**-233**	150
	C2"-OH	**-75**		-256	**-110**	35	**-60**		-484	**-157**	97
	C3"-OH	**-73**		-253	**-103**	30	**-58**		-482	**-146**	88
**3e**	C7"-NH	**-76**	200	-277	**-145**	68	**-84**	418	-502	**-206**	122
	C3"-OH	**-85**		-285	**-119**	35	**-86**		-504	**-179**	93
	C4"-OH	**-83**		-283	**-129**	46	**-84**		-503	**-192**	107
**•OCH$_2$CH$_3$**											
**3a**	C7"-NH	**-79**	228	-307	**-166**	87	**-83**	415	-498	**-224**	140
	C2"-OH	**-41**		-269	**-92**	50	**-29**		-444	**-127**	98
**3b**	C7"-NH	**-77**	217	-294	**-145**	68	**-83**	400	-483	**-198**	115
	C4"-OH	**-51**		-267	**-118**	67	**-48**		-448	**-166**	117
**3c**	C7"-NH	**-78**	221	-278	**-142**	67	**-83**	400	-483	**-197**	114
	C4"-OH	**-59**		-280	**-100**	42	**-54**		-453	**-151**	97
**3d**	C7"-NH	**-76**	183	-259	**-168**	92	**-80**	421	-501	**-227**	148
	C2"-OH	**-73**		-256	**-110**	38	**-57**		-478	**-152**	94
	C3"-OH	**-70**		-253	**-102**	32	**-55**		-476	**-141**	86
**3e**	C7"-NH	**-74**	203	-277	**-145**	71	**-81**	416	-497	**-200**	119
	C3"-OH	**-82**		-285	**-119**	37	**-83**		-499	**-173**	90
	C4"-OH	**-80**		-283	**-128**	48	**-81**		-497	**-186**	105
**•OCH(CH$_3$)$_2$**											
**3a**	C7"-NH	**-85**	222	-307	**-166**	81	**-90**	405	-495	**-221**	130
	C2"-OH	**-48**		-270	**-92**	44	**-36**		-441	**-124**	88
**3b**	C7"-NH	**-83**	211	-294	**-146**	62	**-90**	390	-480	**-195**	105
	C4"-OH	**-57**		-268	**-118**	61	**-55**		-445	**-163**	108
**3c**	C7"-NH	**-85**	215	-278	**-143**	61	**-90**	390	-480	**-194**	104
	C4"-OH	**-65**		-280	**-101**	36	**16**		-450	**-148**	88
**3d**	C7"-NH	**-82**	177	-259	**-168**	86	**-87**	411	-498	**-225**	138
	C2"-OH	**-79**		-256	**-111**	32	**-64**		-475	**-149**	85
	C3"-OH	**-76**		-253	**-103**	26	**-62**		-473	**-138**	76
**3e**	C7"-NH	**-80**	197	-277	**-145**	65	**-88**	406	-494	**-197**	109
	C3"-OH	**-88**		-285	**-119**	31	**-90**		-496	**-171**	81
	C4"-OH	**-87**		-284	**-129**	42	**-88**		-494	**-183**	95
**•OC(CH$_3$)$_3$**											
**3a**	C7"-NH	**-90**	221	-311	**-170**	81	**-95**	401	-496	**-221**	126
	C2"-OH	**-52**		-274	**-96**	44	**-41**		-442	**-125**	85
**3b**	C7"-NH	**-88**	210	-298	**-150**	62	**-94**	386	-481	**-196**	102
	C4"-OH	**-61**		-272	**-122**	61	**-60**		-446	**-163**	104
**3c**	C7"-NH	**-89**	215	-282	**-147**	60	**-95**	386	-481	**-195**	101
	C4"-OH	**-69**		-284	**-105**	35	**-65**		-451	**-149**	84
**3d**	C7"-NH	**-87**	177	-263	**-172**	86	**-91**	407	-499	**-225**	134
	C2"-OH	**-83**		-260	**-115**	31	**-69**		-476	**-150**	81
	C3"-OH	**-81**		-257	**-107**	26	**-67**		-474	**-138**	72
**3e**	C7"-NH	**-84**	197	-281	**-149**	65	**-93**	402	-494	**-198**	105
	C3"-OH	**-93**		-289	**-123**	31	**-94**		-496	**-171**	77
	C4"-OH	**-91**		-288	**-133**	42	**-93**		-495	**-184**	91

Table S2: Thermodynamic parameters for radical scavenging activity of investigated compounds with peroxy radicals (kJ mol^{-1}).

With **peroxy** radicals		**HAT**	**SET-PT**		**SPLET**		**HAT**	**SET-PT**		**SPLET**	
		$\Delta_r G_{HAT}$	$\Delta_r G_{SET}$	$\Delta_r G_{PT}$	$\Delta_r G_{SPL}$	$\Delta_r G_{ET}$	$\Delta_r G_{HAT}$	$\Delta_r G_{SET}$	$\Delta_r G_{PT}$	$\Delta_r G_{SPL}$	$\Delta_r G_{ET}$
		**Water**					**Benzene**				
**•$OOCH_3$**											
**3a**	C7“-NH	**-1**	261	-262	**-122**	120	**-7**	439	-461	**-186**	179
	C2“-OH	**36**		-225	**-47**	83	**48**		-406	**-90**	137
**3b**	C7“-NH	**1**	250	-249	**-101**	101	**-6**	439	-445	**-161**	154
	C4“-OH	**27**		-223	**-73**	100	**29**		-411	**-128**	156
**3c**	C7“-NH	**-1**	255	-234	**-98**	98	**-6**	451	-445	**-160**	153
	C4“-OH	**19**		-236	**-56**	75	**16**		-416	**-114**	137
**3d**	C7“-NH	**2**	216	-214	**-124**	125	**-3**	460	-463	**-190**	187
	C2“-OH	**5**		-211	**-66**	71	**19**		-441	**-114**	133
	C3“-OH	**8**		-209	**-58**	65	**22**		-438	**-103**	125
**3e**	C7“-NH	**4**	236	-232	**-100**	104	**-4**	455	-459	**-162**	158
	C3“-OH	**-4**		-240	**-75**	70	**-6**		-461	**-136**	130
	C4“-OH	**-3**		-239	**-84**	81	**-5**		-459	**-149**	144
**•$OOCH_2CH_3$**											
**3a**	C7“-NH	**-2**	266	-267	**-127**	125	**-6**	456	-462	**-187**	181
	C2“-OH	**36**		-230	**-52**	88	**48**		-408	**-91**	139
**3b**	C7“-NH	**0**	254	-254	**-106**	106	**-5**	441	-447	**-162**	156
	C4“-OH	**27**		-228	**-78**	105	**29**		-412	**-129**	159
**3c**	C7“-NH	**-1**	259	-239	**-103**	104	**-6**	441	-446	**-161**	155
	C4“-OH	**18**		-241	**-61**	79	**24**		-417	**-115**	139
**3d**	C7“-NH	**1**	221	-220	**-129**	130	**-2**	462	-460	**-191**	189
	C2“-OH	**5**		-216	**-71**	75	**20**		-462	**-115**	136
	C3“-OH	**7**		-214	**-63**	70	**22**		-461	**-104**	127
**3e**	C7“-NH	**4**	241	-237	**-105**	109	**-4**	457	-464	**-164**	160
	C3“-OH	**-5**		-245	**-80**	75	**-5**		-442	**-137**	132
	C4“-OH	**-3**		-244	**-89**	86	**-4**		-440	**-150**	146
**•$OOCH=CH_2$**											
**3a**	C7“-NH	**-16**	216	-232	**-92**	75	**-20**	399	-419	**-144**	124
	C2“-OH	**21**		-195	**-17**	38	**34**		-365	**-48**	82
**3b**	C7“-NH	**-15**	204	-219	**-71**	56	**-20**	384	-404	**-119**	101
	C4“-OH	**12**		-193	**-43**	55	**15**		-369	**-86**	99
**3c**	C7“-NH	**-16**	209	-233	**-68**	54	**-20**	383	-403	**-118**	98
	C4“-OH	**4**		-206	**-26**	30	**9**		-374	**-72**	81
**3d**	C7“-NH	**-14**	171	-185	**-94**	80	**-17**	405	-421	**-148**	131
	C2“-OH	**-10**		-181	**-36**	26	**6**		-399	**-72**	78
	C3“-OH	**-8**		-179	**-28**	20	**8**		-397	**-61**	69
**3e**	C7“-NH	**-11**	191	-202	**-70**	59	**-18**	399	-417	**-121**	103
	C3“-OH	**-20**		-210	**-45**	25	**-20**		-419	**-94**	74
	C4“-OH	**-18**		-209	**-54**	36	**-18**		-418	**-107**	89

Table S3: Thermodynamic parameters for radical scavenging activity of investigated compounds with chlorinated methyl-peroxy radicals (kJ mol^{-1}).

With **chlorinated Me-peroxy** radicals		**HAT**	**SET-PT**		**SPLET**		**HAT**	**SET-PT**		**SPLET**	
		$\Delta_r G_{HAT}$	$\Delta_r G_{SET}$	$\Delta_r G_{PT}$	$\Delta_r G_{SPL}$	$\Delta_r G_{ET}$	$\Delta_r G_{HAT}$	$\Delta_r G_{SET}$	$\Delta_r G_{PT}$	$\Delta_r G_{SPL}$	$\Delta_r G_{ET}$
		**Water**					**Benzene**				
**$ClCH_2OO$•**											
**3a**	C7“-NH	**-26**	198	-224	**-83**	58	**-31**	374	-405	**-131**	99
	C2“-OH	**12**		-187	**-9**	21	**23**		-351	**-34**	58
**3b**	C7“-NH	**-24**	187	-211	**-63**	38	**-31**	359	-390	**-105**	75
	C4“-OH	**2**		-184	**-35**	37	**4**		-355	**-73**	77
**3c**	C7“-NH	**-25**	192	-195	**-60**	35	**-31**	359	-390	**-104**	74
	C4“-OH	**-6**		-197	**-18**	12	**-1**		-360	**-58**	57
**3d**	C7“-NH	**-23**	153	-176	**-85**	62	**-28**	380	-408	**-135**	107
	C2“-OH	**-20**		-173	**-27**	8	**-5**		-385	**-59**	54
	C3“-OH	**-17**		-170	**-20**	3	**-3**		-383	**-48**	45
**3e**	C7“-NH	**-21**	173	-194	**-62**	41	**-29**	375	-404	**-107**	78
	C3“-OH	**-29**		-202	**-36**	8	**-31**		-406	**-81**	50
	C4“-OH	**-27**		-200	**-45**	18	**-29**		-404	**-93**	64
**Cl_2CHOO•**											
**3a**	C7“-NH	**-35**	169	-204	**-63**	28	**-39**	343	-381	**-106**	68
	C2“-OH	**2**		-166	**11**	-9	**16**		-327	**-10**	26
**3b**	C7“-NH	**-33**	157	-191	**-42**	9	**-38**	328	-366	**-81**	43
	C4“-OH	**-7**		-164	**-15**	8	**-3**		-331	**-48**	45
**3c**	C7“-NH	**-34**	162	-175	**-39**	5	**-38**	327	-366	**-80**	42
	C4“-OH	**-15**		-177	**3**	-17	**-9**		-336	**-34**	25
**3d**	C7“-NH	**-32**	124	-156	**-65**	33	**-35**	349	-384	**-110**	75
	C2“-OH	**-29**		-153	**-7**	-21	**-12**		-361	**-35**	22
	C3“-OH	**-26**		-150	**-1**	-27	**-10**		-359	**-24**	13
**3e**	C7“-NH	**-30**	144	-174	**-42**	12	**-36**	343	-380	**-83**	47
	C3“-OH	**-38**		-182	**-16**	-22	**-38**		-382	**-56**	18
	C4“-OH	**-36**		-180	**-25**	-11	**-36**		-380	**-69**	33
**Cl_3COO•**											
**3a**	C7“-NH	**-38**	147	-185	**-44**	7	**-41**	315	-356	**-81**	40
	C2“-OH	**-1**		-148	**30**	-31	**47**		-301	**15**	-2
**3b**	C7“-NH	**-36**	136	-172	**-23**	-13	**-40**	300	-340	**-55**	15
	C4“-OH	**-10**		-145	**4**	-14	**-5**		-305	**-23**	18
**3c**	C7“-NH	**-37**	141	-178	**-21**	-16	**-40**	300	-340	**-55**	14
	C4“-OH	**-18**		-158	**21**	-39	**-11**		-311	**-9**	-2
**3d**	C7“-NH	**-35**	102	-137	**-46**	11	**-37**	300	-358	**-85**	48
	C2“-OH	**-32**		-134	**12**	-43	**-15**		-336	**-9**	-5
	C3“-OH	**-29**		-131	**19**	-49	**-12**		-333	**2**	-14
**3e**	C7“-NH	**-33**	122	-155	**-23**	-10	**-40**	316	-354	**-57**	19
	C3“-OH	**-41**		-163	**3**	-44	**-39**		-356	**-31**	-9
	C4“-OH	**-39**		-161	**-6**	-33	**-38**		-354	**-43**	5

Table S4: Thermodynamic parameters for RAF mechanistic pathway of inactivation of $CH_3O^{\bullet}$ by investigated compounds (kJ mol^{-1}).

	Compound	Position	Water	Benzene
$CH_3O^{\bullet}$	**3a**	C5”	25.4	23.0
		C6”	-1.5	0.4
		C7	17.9	15.5
	**3b**	C5”	19.3	16.9
		C6”	18.9	11.7
		C7	17.7	12.0
	**3c**	C5”	28.5	24.7
		C6”	6.0	0.6
		C7	20.6	17.5
	**3d**	C5”	27.1	25.3
		C6”	-9.3	-13.8
		C7	20.5	16.1
	**3e**	C5”	26.7	23.8
		C6”	6.9	2.9
		C7	17.9	14.0

Table S5: Thermodynamic parameters for RAF mechanistic pathway of inactivation of $CH_3CH_2O^{\bullet}$ by investigated compounds (kJ mol^{-1}).

	Compound	Position	Water	Benzene
$CH_3CH_2O^{\bullet}$	**3a**	C5”	30.1	28.4
		C6”	11.2	5.3
		C7	21.1	19.9
	**3b**	C5”	23.4	21.6
		C6”	23.0	16.7
		C7	22.2	17.3
	**3c**	C5”	36.2	30.0
		C6”	15.0	4.7
		C7	24.9	24.9
	**3d**	C5”	35.7	29.8
		C6”	-5.0	-10.4
		C7	22.6	21.5
	**3e**	C5”	33.8	29.4
		C6”	13.8	1.3
		C7	27.2	21.3

Table S6: Thermodynamic parameters for RAF mechanistic pathway of inactivation of $(CH_3)_2CHO^•$ by investigated compounds (kJ mol^{-1}).

	Compound	Position	Water	Benzene
$(CH_3)_2CHO^•$	**3a**	C5"	33.0	29.4
		C6"	6.7	2.4
		C7	23.8	16.8
	**3b**	C5"	19.5	18.1
		C6"	23.1	16.0
		C7	17.3	13.2
	**3c**	C5"	35.3	28.9
		C6"	12.4	6.8
		C7	23.0	16.6
	**3d**	C5"	37.6	33.1
		C6"	-6.8	-10.8
		C7	25.4	19.1
	**3e**	C5"	27.4	27.3
		C6"	12.1	0.8
		C7	21.8	18.2

Table S7: Thermodynamic parameters for RAF mechanistic pathway of inactivation of $(CH_3)_3CO^•$ by investigated compounds (kJ mol^{-1}).

	Compound	Position	Water	Benzene
$(CH_3)_3CO^•$	**3a**	C5"	30.8	26.1
		C6"	17.6	13.3
		C7	35.7	39.8
	**3b**	C5"	38.0	32.6
		C6"	33.0	26.1
		C7	32.8	30.7
	**3c**	C5"	47.8	36.4
		C6"	23.4	16.4
		C7	43.9	39.3
	**3d**	C5"	49.5	44.9
		C6"	2.4	-0.1
		C7	35.5	38.0
	**3e**	C5"	44.7	42.6
		C6"	16.9	18.1
		C7	41.4	37.2

Table S8: Thermodynamic parameters for RAF mechanistic pathway of inactivation of $CH_3COO^•$ by investigated compounds (kJ mol^{-1}).

	Compound	Position	Water	Benzene
$CH_3OO^•$	**3a**	C5”	95.7	87.4
		C6”	73.4	67.5
		C7	89.0	85.7
	**3b**	C5”	85.0	85.6
		C6”	91.2	83.3
		C7	86.2	84.3
	**3c**	C5”	99.9	93.5
		C6”	59.2	68.1
		C7	89.7	90.7
	**3d**	C5”	98.0	94.2
		C6”	59.2	53.6
		C7	89.7	89.8
	**3e**	C5”	104.5	97.0
		C6”	80.8	75.0
		C7	95.2	91.2

Table S9: Thermodynamic parameters for RAF mechanistic pathway of inactivation of $CH_3CH_2OO^•$ by investigated compounds (kJ mol^{-1}).

	Compound	Position	Water	Benzene
$CH_3CH_2OO^•$	**3a**	C5”	91.0	85.9
		C6”	72.6	62.6
		C7	91.8	90.1
	**3b**	C5”	92.6	88.8
		C6”	90.3	82.1
		C7	91.7	88.1
	**3c**	C5”	85.4	80.3
		C6”	79.5	71.5
		C7	96.2	92.6
	**3d**	C5”	94.0	92.1
		C6”	62.6	51.6
		C7	92.0	92.1
	**3e**	C5”	88.6	86.8
		C6”	79.8	70.9
		C7	94.5	92.9

Table S10: Thermodynamic parameters for RAF mechanistic pathway of inactivation of $CH_2=CHOO^•$ by investigated compounds (kJ mol^{-1}).

	Compound	Position	Water	Benzene
$CH_2=CHOO^•$	**3a**	C5”	83.4	82.8
		C6”	63.7	59.2
		C7	73.9	72.6
	**3b**	C5”	82.8	81.7
		C6”	78.6	72.5
		C7	74.6	73.2
	**3c**	C5”	87.8	84.8
		C6”	68.6	61.9
		C7	74.7	82.0
	**3d**	C5”	86.0	85.0
		C6”	48.7	44.3
		C7	74.3	75.1
	**3e**	C5”	86.6	79.7
		C6”	72.2	63.1
		C7	78.1	77.5

Table S11: Thermodynamic parameters for RAF mechanistic pathway of inactivation of $ClCH_2OO^•$ by investigated compounds (kJ mol^{-1}).

	Compound	Position	Water	Benzene
$ClCH_2OO^•$	**3a**	C5”	86.9	81.0
		C6”	66.4	67.1
		C7	56.7	60.7
	**3b**	C5”	86.4	76.6
		C6”	82.8	82.4
		C7	61.5	56.0
	**3c**	C5”	95.5	92.5
		C6”	70.2	60.1
		C7	67.7	72.6
	**3d**	C5”	92.7	81.8
		C6”	57.0	47.8
		C7	61.1	67.8
	**3e**	C5”	88.8	85.4
		C6”	78.0	64.0
		C7	74.1	63.4

Table S12: Thermodynamic parameters for RAF mechanistic pathway of inactivation of $Cl_2CHOO^{•}$ by investigated compounds (kJ mol^{-1}).

	Compound	Position	Water	Benzene
$Cl_2CHOO^{•}$	**3a**	C5”	64.8	55.5
		C6”	45.9	42.0
		C7	64.3	61.1
	**3b**	C5”	54.0	60.7
		C6”	65.5	60.6
		C7	63.4	62.7
	**3c**	C5”	62.8	63.5
		C6”	48.7	43.4
		C7	69.3	60.2
	**3d**	C5”	63.7	69.8
		C6”	30.2	24.2
		C7	65.6	57.2
	**3e**	C5”	68.1	67.2
		C6”	55.7	51.3
		C7	71.0	65.2

Table S13: Thermodynamic parameters for RAF mechanistic pathway of inactivation of $Cl_3COO^{•}$ by investigated compounds (kJ mol^{-1}).

	Compound	Position	Water	Benzene
$Cl_3COO^{•}$	**3a**	C5”	51.6	60.7
		C6”	35.6	33.7
		C7	49.0	49.8
	**3b**	C5”	56.1	57.9
		C6”	51.3	56.1
		C7	55.1	55.4
	**3c**	C5”	63.4	53.9
		C6”	43.8	40.6
		C7	54.9	59.5
	**3d**	C5”	54.9	58.6
		C6”	22.7	19.4
		C7	48.9	61.8
	**3e**	C5”	53.9	56.4
		C6”	40.4	43.5
		C7	55.0	54.8

Table S14: Thermodynamic parameters describing the second step (RAF) of the radical scavenging reaction following the HAT-RAF mechanism in triplet spin state (kJ mol^{-1}).

**Compounds**		**Water**			**Benzene**		
**Triplet**		$(CH_3)_3CO^•$	$CH_2=CHOO^•$	$Cl_3COO^•$	$(CH_3)_3CO^•$	$CH_2=CHOO^•$	$Cl_3COO^•$
**3a**	C5”	35	85	63	32	84	63
	C6”	33	69	45	27	67	44
	C7”	39	85	64	28	87	66
**3b**	C5”	32	86	58	28	85	59
	C6”	46	89	61	45	88	60
	C7”	41	86	68	38	85	67
**3c**	C5”	28	71	39	48	90	62
	C6”	15	56	28	33	77	48
	C7”	22	70	49	43	85	64
**3d**	C5”	33	83	58	34	84	61
	C6”	19	56	30	18	54	30
	C7”	43	87	60	40	85	56
**3e**	C5”	33	88	56	41	88	56
	C6”	32	73	55	31	73	46
	C7”	38	86	68	47	82	68

Table S15: Thermodynamic parameters describing the second step (RAF) of the radical scavenging reaction following the HAT-RAF mechanism in singlet spin state (kJ mol^{-1}).

**Compounds**		**Water**			**Benzene**		
**Singlet**		$(CH_3)_3CO^•$	$CH_2=CHOO^•$	$Cl_3COO^•$	$(CH_3)_3CO^•$	$CH_2=CHOO^•$	$Cl_3COO^•$
**3a**	C5”	-114	-54	-47	-110	-50	-70
	C6”	33	86	67	44	96	78
	C7”	28	104	92	55	69	42
**3b**	C5”	-19	56	41	20	97	80
	C6”	40	100	78	62	114	89
	C7”	42	72	57	11	85	42
**3c**	C5”	-33	40	31	22	90	68
	C6”	-40	15	-5	10	63	48
	C7”	23	52	24	20	68	42
**3d**	C5”	-121	-59	-74	-117	-56	-70
	C6”	-44	8	-12	-43	5	-14
	C7”	25	103	88	54	71	45
**3e**	C5”	-18	62	55	23	100	86
	C6”	-15	42	20	30	67	50
	C7”	40	70	41	23	65	36

Table S16: Gibbs Free Energies of the optimized structures of biradical species (in Hartree)

**Biradical specie**		**Singlet**		**Triplet**	
		Water	Benzene	Water	Benzene
**3a**	C2"-O• and C7"-N•	-1178.33	-1178.32	-1178.35	-1178.34
**3b**	C4"-O• and C7"-N•	-1178.34	-1178.33	-1178.35	-1178.35
**3c**	C4"-O• and C7"-N•	-1292.84	-1292.83	-1292.85	-1292.84
**3d**	C2"-O• and C7"-N•	-1253.56	-1253.55	-1253.59	-1253.58
	C3"-O• and C7"-N•	-1253.61	-1253.60	-1253.59	-1253.58
	C2"-O• and C3"-O•	-1253.61	-1253.60	-1253.55	-1253.55
**3e**	C3"-O• and C7"-N•	-1253.57	-1253.56	-1253.59	-1253.58
	C4"-O• and C7"-N•	-1253.58	-1253.57	-1253.59	-1253.58
	C3"-O• and C4"-O•	-1253.61	-1253.60	-1253.55	-1253.55

Table S17: Drug-likeness rules and bioavailability score

**SwissADME**							
Compounds	Lipinski	Ghose	Veber	Egan	Muegge	Violations	Bio-availability score
**3a**	Yes	Yes	Yes	Yes	Yes	0	0.55
**3b**	Yes	Yes	Yes	Yes	Yes	0	0.55
**3c**	Yes	Yes	Yes	Yes	Yes	0	0.55
**3d**	Yes	Yes	Yes	Yes	Yes	0	0.55
**3e**	Yes	Yes	Yes	Yes	Yes	0	0.55
**ADMET Lab2.0**							
Compounds	Lipinski	Pfizer	GSK	Golden triangle	Natural product-likenees	QED	Synthetic accesbility (SA)Score*
**3a**	Yes	Yes	Yes	Yes	Yes	Too complex	2.339
**3b**	Yes	Yes	Yes	Yes	Yes	Too complex	2.304
**3c**	Yes	Yes	Yes	Yes	Yes	Too complex	2.379
**3d**	Yes	Yes	Yes	Yes	Yes	Attractive	2.495
**3e**	Yes	Yes	Yes	Yes	Yes	Attractive	2.445

*Compounds with the SA score <6 are considered easy to synthetise

Table S18: Predicted pharmacokinetic parameters of investigated compounds by ADMET Lab 2.0 server.

**Compound**	**HIA (GI)**	**BBB**	**Log*Kp* (cm/s)**	**P−gp substrate**	**CYP1A2 inhibitor**	**CYP2C19 inhibitor**	**CYP2C9 inhibitor**	**CYP2D6 inhibitor**	**CYP3A4 inhibitor**
**3a**	>30%	No	−5.17	No	Yes	No	Yes	No	No
**3b**	>30%	No	−5.06	No	Yes	No	Yes	No	No
**3c**	>30%	No	−5.20	No	Yes	No	Yes	No	Yes
**3d**	>30%	No	−5.65	No	Yes	No	Yes	No	Yes
**3e**	>30%	No	−5.57	No	Yes	No	No	No	Yes

Table S19: HOMO orbital energies of investigated biradical species in triplet and singlet spin states.

Compound		HOMO	
		Spin state	
	**Positions**	Singlet	Triplet
**3a**	C2”-O• and C7”-N•	-0.28378	-0.26117
**3b**	C4”-O• and C7”-N•	-0.28115	-0.26775
**3c**	C4”-O• and C7”-N•	-0.26872	-0.26413
**3d**	C2”-O• and C7”-N•	-0.2778	-0.25316
	C3”-O• and C7”-N•	-0.27959	-0.28172
	C2”-O• and C3”-O•	-0.23737	-0.3041
**3e**	C3”-O• and C7”-N•	-0.27645	-0.26053
	C4”-O• and C7”-N•	-0.27335	-0.26439
	C3”-O• and C4”-O•	-0.23623	-0.30339

Table S20: HAT-RC mechanism of radical scavenging activity for compounds **3a-3e** (kJ mol^{-1}).

**Compound**	Water			Benzene		
	$(CH_3)_3CO^\bullet$	$CH_2=CHOO^\bullet$	$Cl_3COO^\bullet$	$(CH_3)_3CO^\bullet$	$CH_2=CHOO^\bullet$	$Cl_3COO^\bullet$
**3a**	-81	-30	-53	-83	-33	-54
**3b**	-86	-39	-63	-88	-35	-61
**3c**	-83	-29	-58	-87	-34	-61
**3d**	-79	-27	-53	-81	-28	-54
**3e**	-84	-33	-64	-86	-35	-67

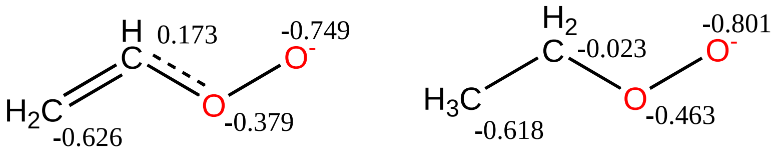

Figure S1: NBO charge distribution of $CH_2=CHOO^-$ (left) and $CH_3CH_2OO^-$ (right)

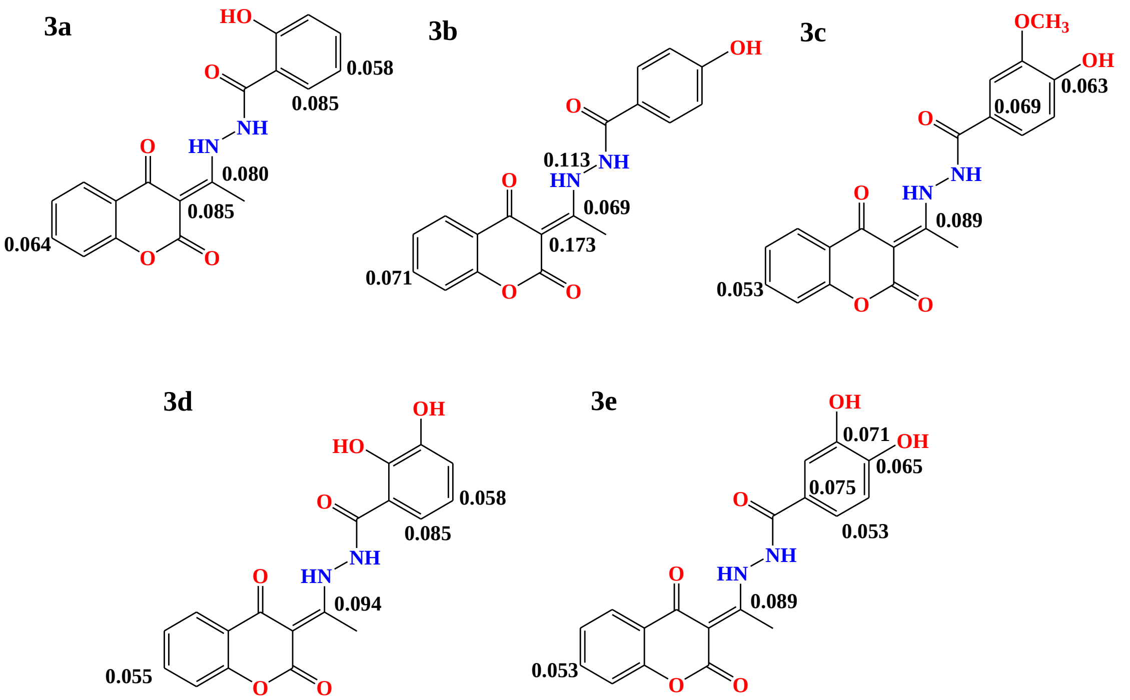

Figure S2: Fukui index values for investigated compounds with values higher than 0.05 presented

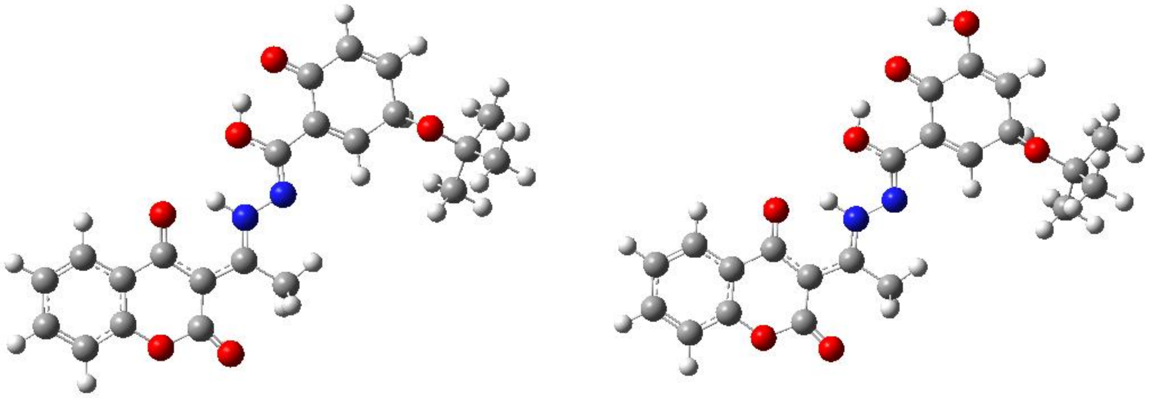

3aC5″-OC(CH3)3 **3d**C5″-OC(CH3)3

Figure S3: Optimised structures of the compounds stabilised by hydrogen transfer from C2″-OH to C7″=O

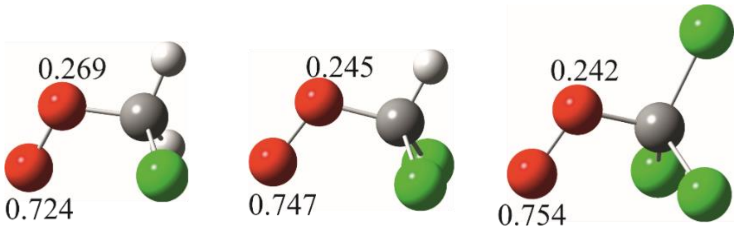

Figure S4. NBO spin distribution on the oxygen atoms of chlorinated methyl radicals

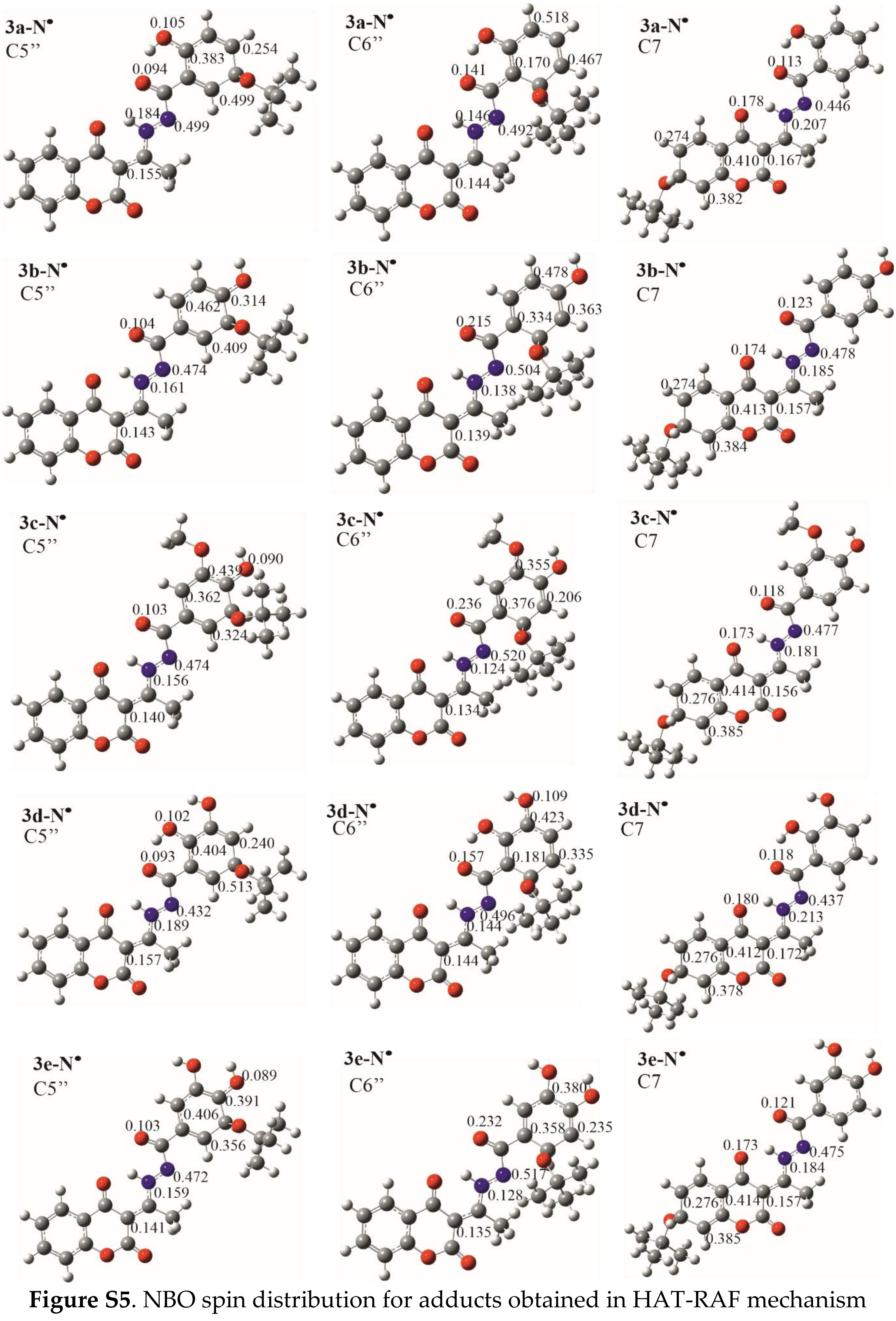

Figure S5. NBO spin distribution for adducts obtained in HAT-RAF mechanism with $(CH_3)_3CO^{\bullet}$
